# *Parotocinclus nandae*, a new distinctive colored catfish (Loricariidae: Hypoptopomatinae) from the upper Rio Paraguaçu, Bahia State, northeastern Brazil

**DOI:** 10.1371/journal.pone.0236690

**Published:** 2020-07-31

**Authors:** Pablo Lehmann A., Priscila Camelier, Angela Zanata

**Affiliations:** 1 Laboratório de Ictiologia, Universidade do Vale do Rio dos Sinos, São Leopoldo, Rio Grande do Sul, Brazil; 2 Programa de Pós-Graduação em Biodiversidade e Evolução, Instituto de Biologia, Universidade Federal da Bahia, Salvador, Bahia, Brazil; Pontificia Universidade Catolica do Rio Grande do Sul, BRAZIL

## Abstract

A new species of *Parotocinclus* from the upper Rio Paraguaçu, Bahia, Brazil, is described. The new species is distinguished from all congeners by its unique color pattern, with irregular dark blotches resulting in a somewhat marble-spotted pattern on head and trunk of most specimens and dorsum of head with a conspicuous V-shaped light mark from tip of snout to nares. The new species is also distinguished from congeners by having the lower lip elongated posteriorly and reaching or surpassing the anterior margin of cleithrum on the pectoral girdle, the canal cheek plate on the ventral surface of the head reduced and with a slightly concave margin, and abdomen covered by small embedded platelets, without contact with each other and not arranged in a line between the pectoral-fin axilla and pelvic-fin origin. The presence of a thick and rough skin in the interradial membrane of pelvic fin exclusively in the females of *P*. *nandae* is reported by the first time to occurs in Siluriformes.

## Introduction

The loricariid fishes of the subfamily Hypoptopomatinae, or small armored catfishes known as “cascudinhos” in Brazil, includes 32 valid genera and 247 species distributed throughout most of lowland tropical South America and generally found in shallow streams and typically associated with emergent and marginal macrophytes [1, 2]. *Parotocinclus* Eigenmann & Eigenmann, 1889, one of the most species-rich genus of Hypoptopomatinae, includes 34 valid species with standard length ranging from 20 to 80 mm and distributed across cis-Andean South America from Colombia and Venezuela to northern Argentina [3–7]. The polyphyletic nature of *Parotocinclus* has been demonstrated by morphology- and DNA-based phylogenetic studies (e.g., Gauger & Buckup [8]; Lehmann [9]; Cramer et al. [10]; Roxo et al. [11]; Pereira & Reis [12], and Reis et al. [13]), but the persistent recognition of this genus in spite of its polyphyletic nature is maintained by the possession of an adipose fin, which is unique among the genera of the subfamily [4].

The type species of the genus, *Parotocinclus maculicauda* (Steindachner, 1877), from coastal rivers situated between Rio de Janeiro and Santa Catarina states, was described in Steindachner [14] and according to Lehmann [9] apparently is closely related to *P*. *doceanus* (Miranda Ribeiro, 1918), *P*. *fluminense* Roxo, Melo, Silva & Oliveira, 2017 (= *Parotocinclus* sp. n. 7 of Lehmann [9]), and *P*. *jimi* Garavello, 1977, all species distributed south of the Rio São Francisco [14–17].

Recent expeditions in coastal drainages of Bahia State revealed a new loricariid genus (*Hirtella* Pereira, Zanata, Cetra & Reis, 2014 [18]) and a series of new loricariid species of the genera *Hypostomus* Lacépède, 1803, *Pareiorhaphis* Miranda Ribeiro, 1918, and *Parotocinclus* [15, 19, 20]. At least four of these species are from the Paraguaçu basin (i.e., Birindelli et al. [21]; Zanata et al. [22]; Pereira & Zanata [23]; Pereira et al. [20]), suggesting that this drainage is still highly unexplored and reinforcing the endemic nature of its ichthyofauna.

A new species of *Parotocinclus* from Northeastern Mata Atlântica freshwater ecoregion (Abell et al. [24]), the seventh species of loricariid known from the Rio Paraguaçu basin, is described herein. Extensive ichthyological surveys throughout coastal rivers of northeastern Brazil failed to record the new species outside the upper Rio Paraguaçu basin, rendering the species apparently endemic to this drainage.

## Material and methods

The live specimens were anesthetized in a Eugenol solution dissolved in ethyl alcohol in 1:9 ratio (clove oil: ethyl alcohol), and this solution was then diluted with water in order to obtain concentrations of 0.20 mL of clove oil per 500 mL of water. Posteriorly, the specimens were fixed in 10% formalin, and then transferred to 70% ethanol. This protocol has been approved by the commission of ethical use of animals of the Universidade do Vale do Rio dos Sinos (CEUA/UNISINOS) which considerers animal welfare laws. Permission for collecting was granted by ICMBio # 13754–1. Comparative material of *Parotocinclus* species used herein are those listed in Lehmann [9], Lehmann & Reis [25], and Lehmann et al. [26]. Counts and measurements followed Lehmann et al. [6]. Measurements were taken as point-to-point linear distances with digital calipers under a dissecting scope on the left side of individuals, recorded to the nearest 0.1 mm, following mainly Boeseman [27] and Schaefer [1], and expressed as percents of standard length (SL) or head length (HL). Identification and counts of dermal plates follow the serial homology scheme proposed by Schaefer [1]. Counts are given in Table 1. For the anatomical analyses, specimens were cleared and stained (c&s) for visualization of bones and cartilages following the protocol of Taylor & Van Dyke [28]. Dermal plates and vertebral centra were counted from c&s material. Vertebral counts include the five centra in the Weberian Apparatus and the fused ural +preural centra are counted as one element. llustrations were prepared under a stereomicroscope with a camera lucida and edited electronically. Type specimens and comparative material are deposited in the Laboratório de Ictiologia, Universidade do Vale do Rio dos Sinos, UNISINOS, São Leopoldo (UNICTIO), Museu de Ciências e Tecnologia da Pontifícia Universidade Católica do Rio Grande do Sul, Porto Alegre (MCP), Museu de Zoologia da Universidade de São Paulo, São Paulo (MZUSP), and Museu de História Natural da Bahia, Universidade Federal da Bahia, Salvador (UFBA).

**Table 1:** Morphometric and meristic data of holotype and 20 paratypes of *Parotocinclus nandae*. Measurement values given as percent of standard length or head length. SD = standard deviation, H = holotype.

	H	Low	High	Mean/Mode*	SD
Standard length (mm)	39.8	33.1	43.8	38.6	-
**Percent of standard length**					
Head length	29.0	28.5	33.7	31.5	1.3
Pre-dorsal length	41.2	40.7	44.1	42.3	1.1
Pre-pectoral distance	24.0	24.0	27.8	25.7	0.8
Pre-anal length	55.5	55.5	60.4	58.1	1.4
Pre-adipose length	72.5	71.7	78.3	74.4	1.5
Dorsal-fin spine length	17.3	17.3	20.4	19.2	0.8
Anal-fin unbranched ray length	14.3	14.1	17.1	15.8	0.8
Pectoral-fin spine length	17.5	16.8	20.2	18.5	0.8
Ventral-fin unbranched ray length	17.0	14.8	20.4	17.6	1.8
Upper principal caudal-fin ray	23.6	23.2	29.0	25.1	1.6
Lower principal caudal-fin ray	24.1	24.1	29.9	27.1	1.6
Adipose-fin spine length	8.3	6.3	9.9	8.1	0.9
Adipose to caudal fin distance	24.2	20.2	24.5	22.8	1.2
Abdominal length	20.1	20.1	22.9	21.7	0.9
Cleithral width	21.3	21.2	23.8	22.4	0.7
Trunk depth at dorsal-fin origin	13.6	11.8	15.5	14.0	0.7
Caudal peduncle length	35.1	29.0	37.8	34.7	2.0
Caudal peduncle depth	7.7	6.6	8.2	7.6	0.3
**Percent of head length**					
Snout length	55.5	51.0	61.9	54.0	2.5
Orbital diameter	14.4	12.5	16.4	14.0	1.0
Interorbital width	36.0	31.5	39.4	34.0	2.1
Head depth	45.7	38.9	49.6	42.6	2.5
Premaxillary ramus left	11.3	8.0	13.8	11.0	1.3
Dentary ramus right	9.8	5.7	10.8	8.9	1.1
**Counts**					
Left premaxillary teeth	20	15	23	20*	
Right premaxillary teeth	21	15	25	20*	
Left dentary teeth	17	11	21	16*	
Right dentary teeth	17	12	21	15*	
Plates in median lateral series left	25	24	27	26*	
Plates between dorsal and adipose	6	6	7	6*	
Plates between adipose and caudal	7	6	7	7*	
Plates at anal-fin base	3	3	3	3*	
Plates between anal and caudal fins	11	11	11	11*	
Preadipose azygous plates	1	1	1	1*	
Plates in dorsal series	24	24	25	25*	
Plates in mid-dorsal series	15	15	16	15*	
Plates in mid-ventral series	18	18	20	18*	
Plates in ventral series	21	20	21	21*	

## Results

**Fig 1:**
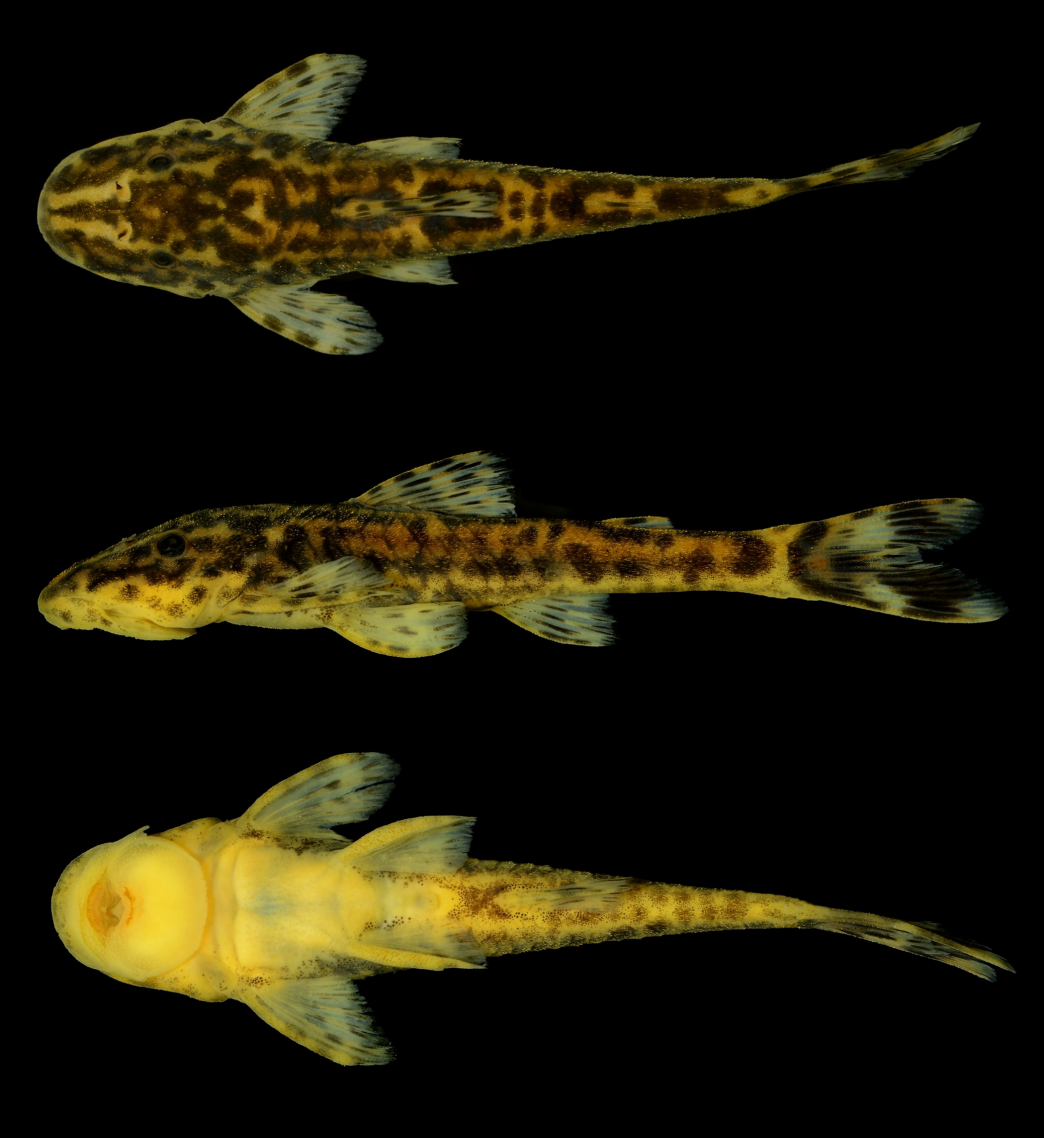
*Parotocinclus nandae*, holotype. MCP 54184, 39.8 mm SL, male; Brazil, Bahia, Ibicoara, upper Rio Paraguaçu, under bridge on highway BA-142, between Ibicoara and Barra da Estiva, 13°26'10.32"S, 41°20'19.14"W.

*Parotocinclus nandae* sp. nov. urn:lsid:zoobank.org:act:B6A4F8E2-68C3-4E45-9D12-6C2138B6B7B4 (Fig 1).

### Holotype

MCP 54184, 39.8 mm SL, male; Brazil: Bahia: Ibicoara, upper Rio Paraguaçu, under bridge on highway BA-142, between Ibicoara and Barra da Estiva (9 km from Ibicoara), 13°26'10.32"S, 41°20'19.14"W, 1068 m above sea level; 9 Jul 2011; A. Zanata, P. Camelier, J. L. O. Birindelli, R. Burger & B. Sardeiro.

### Paratypes

MCP 54185, 8, 33.7–41.5 mm SL (2 c&s, 40.1–41.3 mm SL). UNICTIO 2967, 10, 33.1–43.8 mm SL; MZUSP 125264, 8, 34.7–43.1 mm SL; and UFBA 6976, 7, 35.0–40.7 mm SL; collected with holotype. MZUSP 125265, 4, 35.4–46.2 mm SL; and UFBA 8423, 4, 36.8–45.2 mm SL; 18 Jun 2017, A. Zanata, R. Burger & G. Oliveira. UFBA 7362, 10, 1 tissue sample, 36.6–40.2 mm SL; same locality as holotype 1 May 2013, A. Zanata, R. Burger, L. Oliveira & R. Abreu. MZUSP 120536, 5, 28.9–31.8 mm SL; Brazil: Bahia: Lençóis, Rio Capivari, tributary of Rio São José, Rio Paraguaçu basin, 12°37'27.00"S 41°22'35.70"W; 1 Nov 2015; M. R. S. Melo, A. Clistenes, P. E. S. Moura, E. Santos & C. Santos.

### Diagnosis

**Fig 2:**
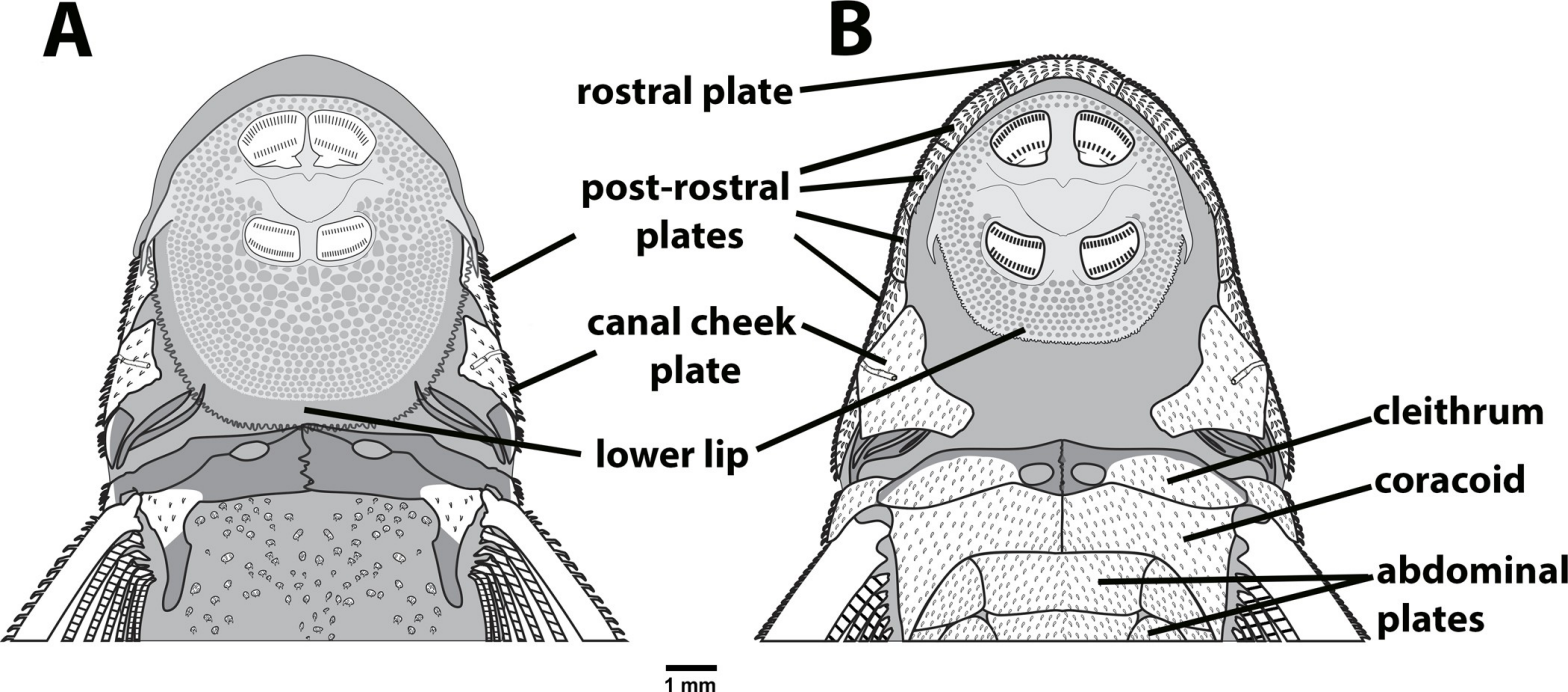
Schematic drawing of the ventral side of head and pectoral girdle, (A) *Parotocinclus nandae*, UNICTIO 2967, 43.7 mm SL, showing the lower lip elongated posteriorly reaching to the pectoral girdle and the canal cheek plate reduced. (B) *P*. *maculicauda*, MCP 31591, 50.4 mm SL, with the lower lip not elongated and the canal plate expanded mesially and posteriorly.

The new species is distinguished from all congeners by its unique color pattern, with irregular dark blotches resulting in a somewhat marble-spotted pattern on head and trunk, and dorsum of head with a conspicuous pale V-shaped mark extending from tip of snout to, or slightly posterior of, nares (Fig 1). *Parotocinclus nandae* can be further diagnosed from its congeners by having the lower lip elongated posteriorly, longer than wide, and reaching to or surpassing the anterior margin of cleithrum (*vs*. lower lip not elongated, wider than longer, and falling distinctly short of pectoral girdle); canal cheek plate on the ventral surface of the head not expanded mesially or posteriorly, with a slightly concave margin (*vs*. canal cheek plate expanded mesially or posteriorly, with triangular tip) (Fig 2); and abdomen covered by small embedded platelets not in contact with each other and not aligned between pectoral- and pelvic-fin origins (*vs*. abdomen completely lacking plates or covered with plates usually contacting each other and arranged in transverse lines).

### Description

**Fig 3:**
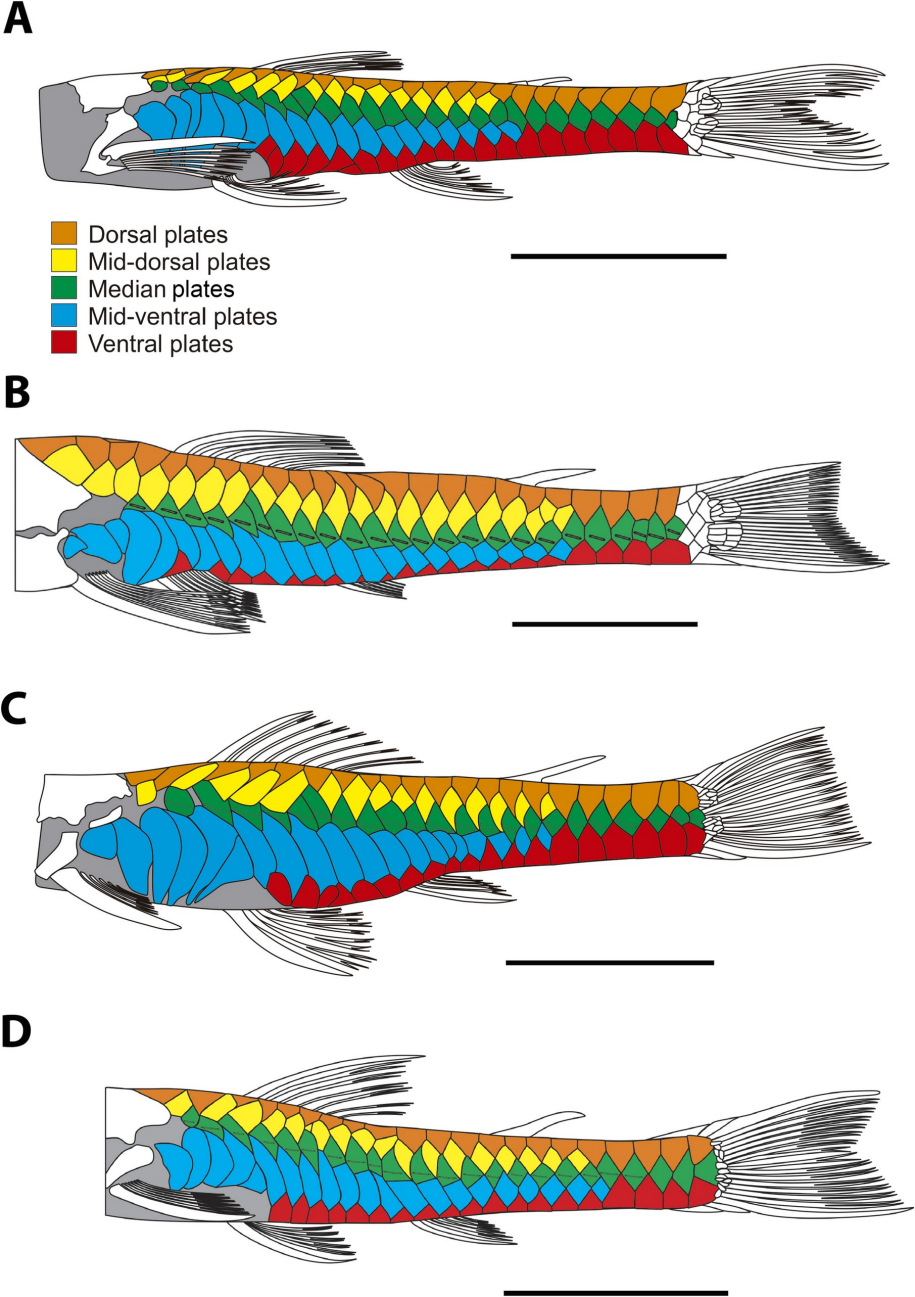
Dermal plates of the lateral trunk and serial plate homology of *Parotocinclus*. (A) *Parotocinclus nandae*, paratype, MCP 54185, 41.3 mm SL; (B) *P*. *adamanteus*, UFBA 51.3 mm SL; (C) *P*. *spilosoma*, MCP 39165, 37.5 mm SL; (D) *P*. *jimi*, UFBA 3869, 36.6 mm SL; Scale bar 1 cm.

Proportional measurements and counts in Table 1. Adult size medium to large for members of genus (maximum size of adult females46.2 mm in SL, MZUSP 125265). Body elongate, without conspicuous keels, moderately depressed. Caudal peduncle vertically oval in cross section. Dorsal profile of head from snout tip to posterior region of parieto-supraoccipital slightly convex. Dorsal profile of trunk straight to slightly concave from tip of parieto-supraoccipital process to adipose-fin origin and slightly concave from adipose-fin base to caudal fin. Greatest trunk depth at dorsal-fin origin. Least trunk depth at shallowest part of caudal peduncle, anterior to procurrent caudal-fin rays. Ventral profile slightly concave from snout tip to cheek canal plate, straight to slightly convex from that point to anus, slightly slanted from anus to end of anal-fin base, almost straight along caudal peduncle. Lateral-line canal complete and uninterrupted, with pored tubes visible from compound pterotic to penultimate plate in median lateral series. Mid-dorsal and mid-ventral series of lateral plates incomplete, terminating 7–8 plates before caudal fin (Fig 3A). Dorsal surface of trunk covered by plates except for small naked area at opening of swimbladder capsule, posteroventrally to compound pterotic. Large area in ventral surface of head naked. Canal cheek plate on ventral surface of head covered partially by skin and partially exposed supporting odontodes. Canal bearing lateral cheek plate with unbranched canal and not expanded mesially and posteriorly without triangular tip, instead, with slightly concave margin, not contacting cleithrum. Head moderately deep, slightly oval in dorsal view. Interorbital space flat to slightly convex; superior margin of orbit not elevated. Parieto-supraoccipital gently elevated posterior to orbit. Snout straight, tip gently convex in lateral profile. Snout tip with extensive area of naked skin, connected ventrally to upper lip, devoid of odontodes. Rostral plate not exposed on the tip of snout and ventrally. Five to seven post-rostral plates along lateral margin of snout with odontodes slightly enlarged, curved and posteriorly oriented. Post-rostral plates not exposed ventrally, except by posterior post-rostral plate adjacent to canal bearing lateral cheek plate. Snout plates anterior to nostrils forming four paired plates. Nostrils ovoid and closer adjacent to anterior margin of orbit than snout. Preopercle exposed and with odontodes laterally. Eye small, dorsolaterally placed. Iris with small dorsal operculum. Internareal area slightly elevated. Posterior tip of parieto-supraoccipital with small patch of odontodes only slightly enlarged relative to those of remainder of head and predorsal area. Abdomen with small embedded platelets, without contacting each other and not arranged in line between pectoral-fin axilla and pelvic-fin origin; plates bearing small odontodes; some specimens with higher concentration of plates between pelvic fins. Specimens up to 35 mm SL with nearly naked areas on abdomen and few irregularly arranged platelets or groupings of tiny odontodes. Pre-anal region with few somewhat large rounded plates not contacting each other, distributed irregularly in mosaic pattern. Pectoral girdle exposed laterally and covered by thick skin medially; odontodes restricted to coracoid. Arrector fossa near symphysis of pectoral girdle and moderately open medially. Coracoid with foramen for passage of arrector ventralis muscle rounded and open laterally.

Oral disk ovoid. Lips well developed, occupying entire ventral surface of head, and with fringed edges and densely covered by papillae; lower lip elongated posteriorly, longer than wide, reaching or surpassing cleithrum, usually longer in larger specimens, with larger papillae immediately posterior to dentaries, decreasing in size posteriorly. Posterior border of lower lip slightly fringed. Maxillary barbel short, mostly adnate to lower lip and with free distal portion similar in size to pupil diameter. Teeth small and delicate; asymmetrically bifid. Medial cusp large and wide, slightly rectangular, lateral cusp small and pointed, reaching up to one-third of medial cusp. Accessory patch of unicuspid teeth absent from both premaxillary and dentary bones.

**Fig 4:**
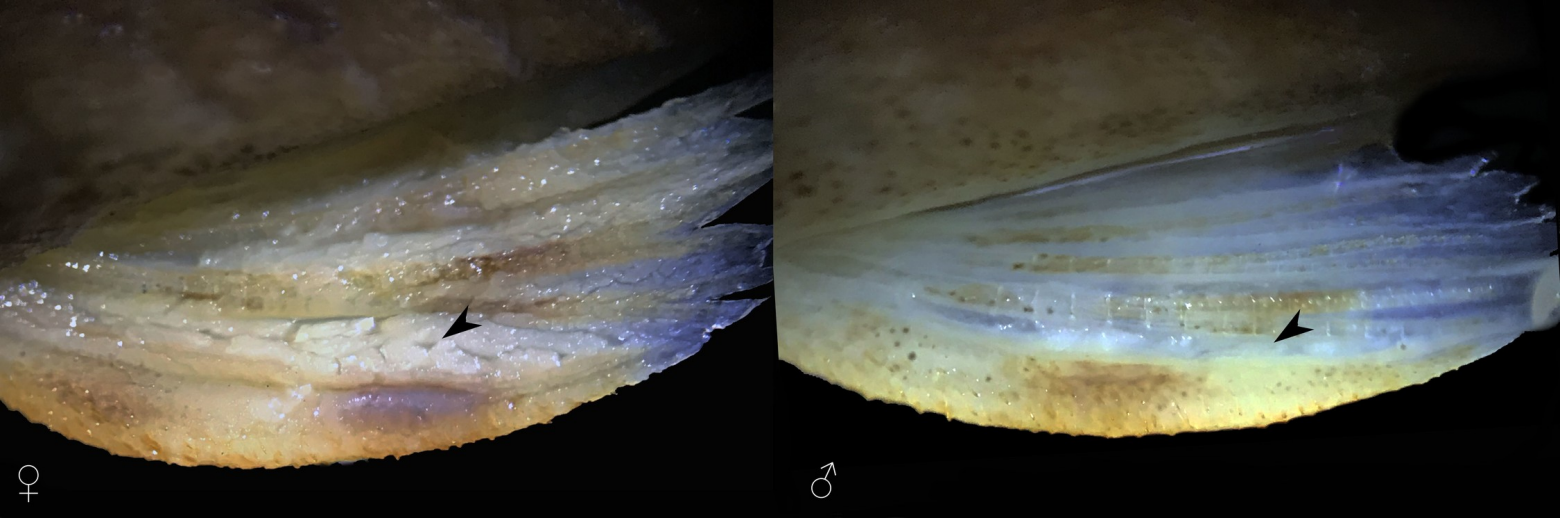
*Parotocinclus nandae*, thick skin covering dorsal surface of the pelvic-fin at interradial membranes. MCP 54185, paratypes, Brazil, Bahia, Ibicoara, upper Rio Paraguaçu basin: (Left) female, 41.5 mm SL; (Right) male, 40.9 mm SL.

Dorsal fin originating at or slightly anterior to vertical line passing through end of pelvic-fin base. Nuchal plate exposed, not covered by skin. Dorsal-fin spinelet plate-like, trapezoidal in shape, wider anteriorly. Dorsal-fin locking mechanism nonfunctional. Dorsal fin short, its base flanked by 5–6 plates; tip of adpressed fin reaching third series of plates behind its base. Dorsal-fin spine moderately flexible, followed by seven branched rays. Distal margin of dorsal fin straight. Adipose fin present, well-ossified and covering three dorsal plates when adpressed; leading spine bearing odontodes and preceded by one middorsal, azygous plate. Pectoral fin moderate in size, with slightly curved and flattened spine and six branched rays; first and second branched rays distinctly longer than spine and subsequent branched rays decreasing gradually in size. Posterior margin of pectoral fin slightly rounded between spine, first, and second branched rays, and straight between subsequent branched rays. Pectoral fin overlapping one third to half-length of pelvic fin when adpressed. Pectoral fin axillary slit absent. Pelvic fin with one unbranched and five branched rays; posterior tip of adpressed fin almost reaching to or just passing anal-fin origin in males, not reaching this point in females (see holotype, Fig 1). Pelvic-fin unbranched ray thickened, slightly depressed and curved, covered with small odontodes ventrally and laterally; odontodes on ventral surface of ray strongly turned mesially. Males with developed dermal flap along postero-dorsal margin of unbranched pelvic-fin ray, distinctly higher near fin base; flap absent in females. Thick skin covering dorsal surface of the pelvic-fin at interradial membranes, more developed in females than males (Fig 4). Anal fin with one unbranched and five branched rays; tip of adpressed fin reaching seven plates behind its base. Caudal-fin posterior margin concave or slightly forked; lower lobe slightly longer than upper; 14 branched rays. Upper caudal-fin lobe with 4 or 5 and lower lobe with 3 or 4 plate-like procurrent rays. Vertebral centra 30 (2 c&s). Five paired pleural ribs (vertebrae 8–12), two associated with connective tissues of vertebrae 8 and 9 (2 c&s).

### Color in alcohol

**Fig 5:**
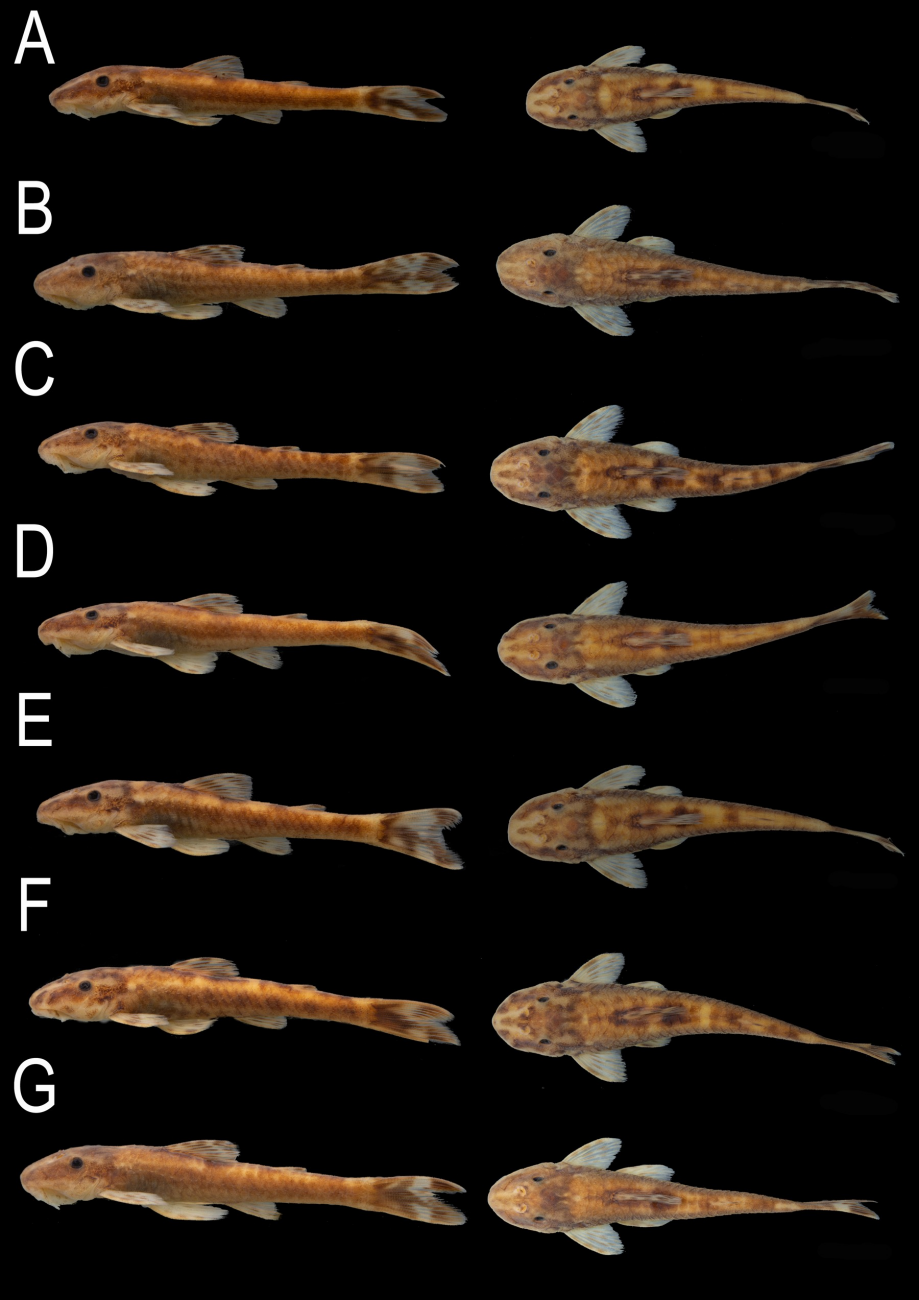
*Parotocinclus nandae*, variation in color pattern in alcohol. MCP 54185, paratypes, Brazil, Bahia, Ibicoara, upper Rio Paraguaçu basin: (A) male, 38.1 mm SL; (B) female, 37.8 mm SL; (C) female, 39.5 mm SL, (D) female, 41.2 mm SL; (E) female 41.2 mm SL; (F) female, 41.7 mm SL; and (G) male, 42.0 mm SL.

Overall ground color of head and trunk yellowish or whitish beige. Dorsal surface of head with conspicuous V-shaped light mark, extending throughout snout tip, rostral plates, nares, frontal, crossing sphenotic, and compound pterotic; V-shaped mark more conspicuous from snout tip to nares in most specimens. Ventral surface of head whitish tan, with few melanophores throughout lateral and anterior margin of lips. Surface of lips pale yellow. Dorsolateral region of head, predorsal area, and dorso-lateral surface of trunk usually with irregular dark blotches forming somewhat marble-spotted color pattern; blotches varying from rounded and isolated from each other to partially merged forming larger connected blotches; some specimens with trunk somewhat homogeneously dark overall, without visible blotches. Dark wide and inconspicuous midlateral stripe initiating on rear of head and usually less evident on posterior portion of trunk; dorsal and ventral margins of stripe mostly irregular, usually merged with blotches dorsally. Trunk ventral of dark stripe usually less pigmented than dorsal portion, but some individuals with trunk homogeneously darkened overall. Ventral surface of trunk uniformly yellowish tan, without dark spots on central portion but with scattered patches of melanophores laterally between pectoral- and pelvic-fin insertions, and from area around anus to end of caudal peduncle. Dorsal, pectoral, pelvic, and anal fins with dark dashes on rays, sometimes forming bands crossing fins; interradial membranes usually hyaline; darkly pigmented individuals usually with fins also more homogeneously darkened and dark dashes less visible. Dark bands on dorsal fin variably positioned, irregularly disposed or forming two oblique transverse bands, one at midlength of rays and other more distally positioned. Pectoral and pelvic fins with dashes irregularly distributed or forming up to three transverse bands. Anal fin with one or two dark bands crossing fin. Adipose fin with one or two dark patches of melanophores; membrane usually hyaline. Caudal fin with variable position of dark and clear areas, but usually with anterior third of fin and distal portions of rays black; most specimens with either vertical light band crossing midlength of fin, or rounded clear areas at midlength of lobes (Figs 1 and 5).

### Color in life

**Fig 6:**
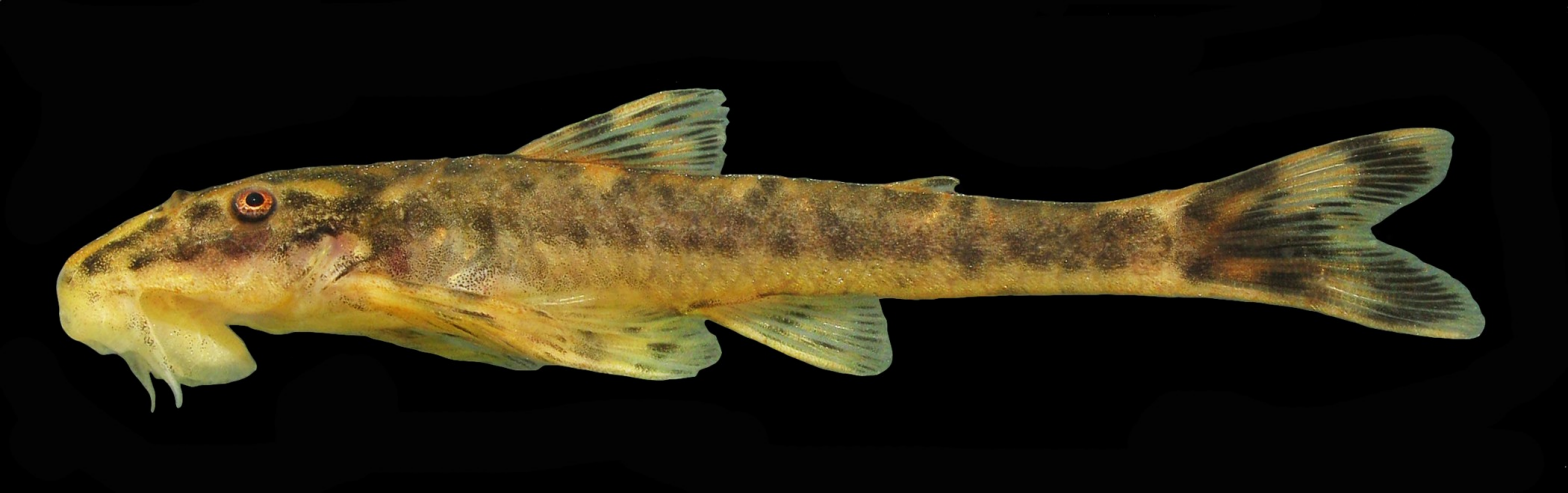
Color in life of *Parotocinclus nandae* in left lateral view. UFBA 6976, paratype, 40.7 mm SL, Brazil, Bahia, Ibicoara, upper Rio Paraguaçu basin, 13°26'10.32"S, 41°20'19.14"W.

Pattern of dark blotches described in preserved material even more evident in freshly collected specimens, distributed on light yellow background. Dorsal half of head and trunk with golden areas, mainly between dorsolateral black blotches. Borders of lips and distal portion of barbels hyaline (Fig 6).

### Sexual dimorphism

Males of *Parotocinclus nandae* possess a conical urogenital papilla, positioned just behind the anal opening; papilla absent in females. Males also possess a developed dermal flap along the postero-dorsal margin of the first pelvic-fin unbranched ray, distinctly higher near fin base; flap absent in females. The pelvic fin is longer in males than in females; posterior tip of adpressed pelvic fin of males reaches to or surpasses anal-fin origin. The interradial membrane of pelvic fin of males is thin and translucent and falls short of that point in females, while in females it is thicker with opaque aspect and rough texture (Fig 4). Furthermore, females reach a larger size than males (33.7–46.2 mm SL, mean 39.6 *vs*. 33.1–40.9 mm SL, mean 37.6; respectively).

### Geographic distribution

**Fig 7:**
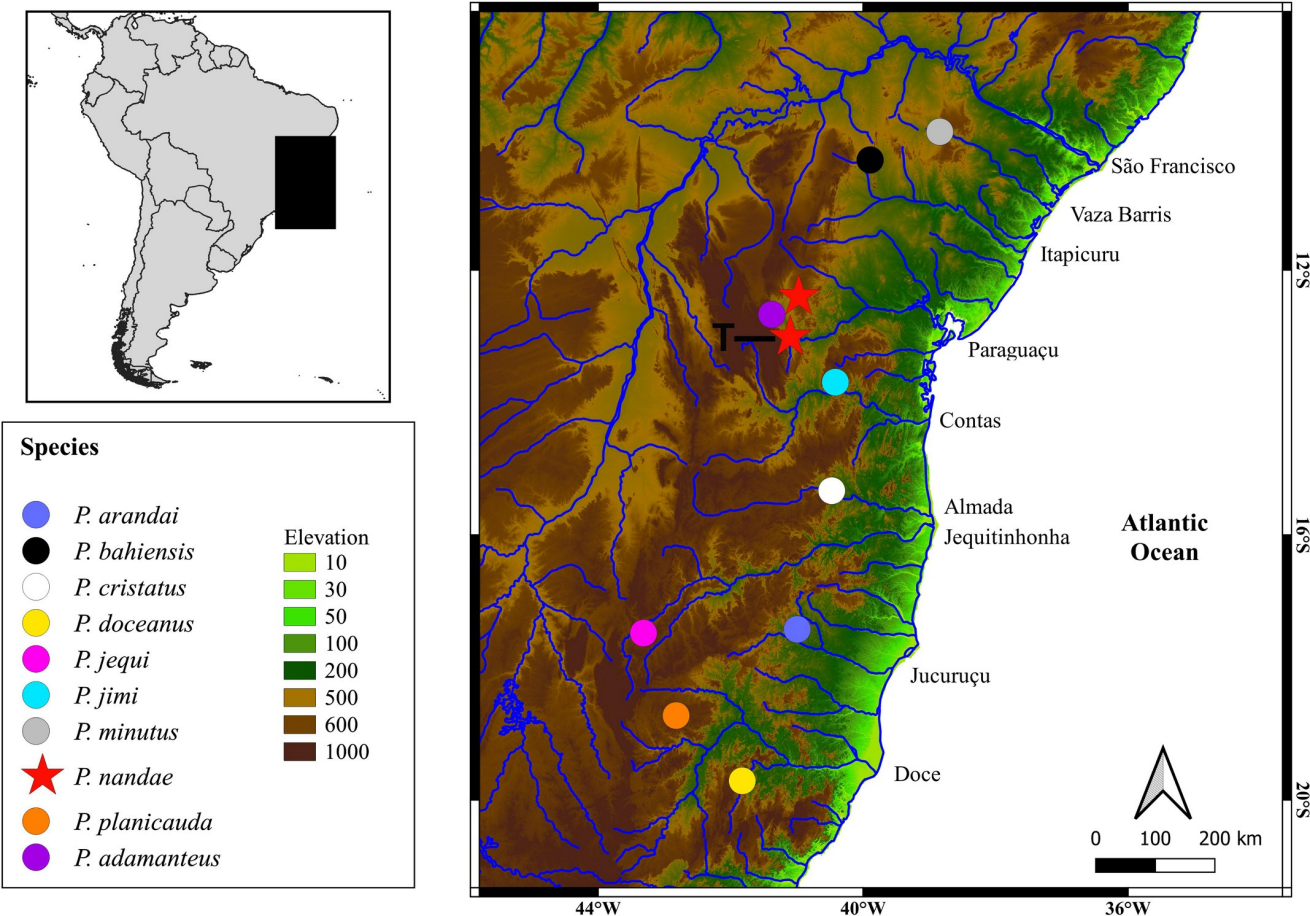
Northeastern Mata Atlântica ecoregion of Brazil, highlighting the type localities of *Parotocinclus* species in this region. **Red stars represent the localities where *Parotocinclus nandae* occurs.** T = type-locality.

*Parotocinclus nandae* is known from two localities in the upper portion of the Rio Paraguaçu basin, Chapada Diamantina domain, Bahia State, Brazil (Fig 7).

### Etymology

The specific name *nandae* honors Maria Fernanda Boaz Lehmann, daughter of the first author of this paper and affectionately known as “Nanda”. A noun in genitive.

### Habitat and ecological notes

**Fig 8:**
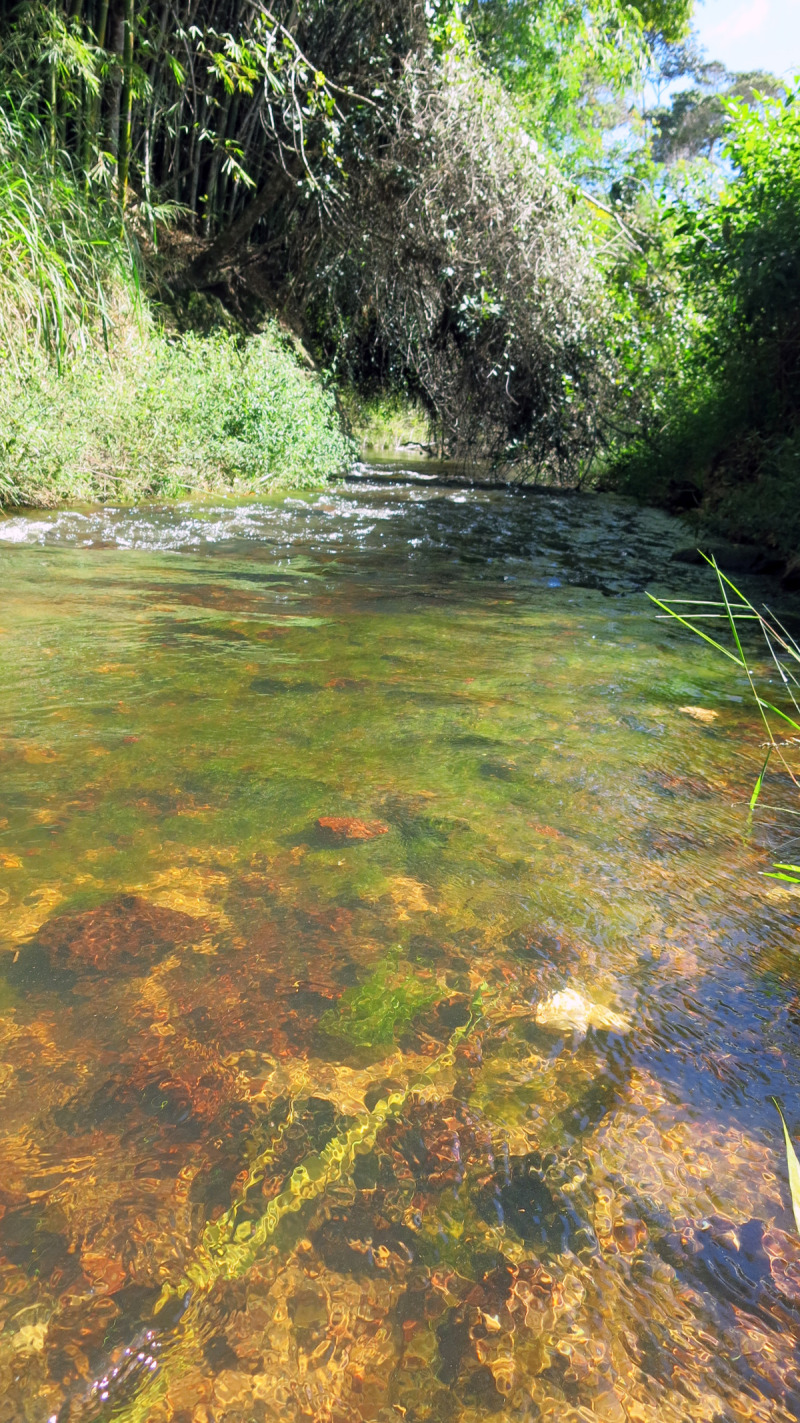
Type locality of *Parotocinclus nandae*. Brazil, Bahia State, Ibicoara, upper Rio Paraguaçu.

The new taxon is known from two localities in the upper Rio Paraguaçu basin, Bahia, Brazil (Fig 8). In the type locality the Rio Paraguaçu is a small clear water stream, with alternating stretches of fast current and backwater, and riparian vegetation mainly composed of shrubs and trees. The specimens of *P*. *nandae* were captured in the fast current portion of the river, in a stretch approximately 2–4 m wide and 0.3 to 1.0 m deep, with pebbles and organic debris composing the substrate and supporting a large amount of subaquatic vegetation. *Astyanax hamatilis* Camelier & Zanata, 2014 and *Rhamdia* sp. were the only species collected syntopically in this locality. The second locality is a stretch of the Rio Capivari, in the municipality of Lençóis, approximately 160 km from the type locality in a straight line. Environmental data of this locality is unavailable. The congener *P*. *adamanteus* was sampled syntopically in this location. *Parotocinclus nandae* is apparently endemic from the Paraguaçu river basin. The possible strongly endemic nature of the Rio Paraguaçu ichthyofauna was already recorded by Zanata et al. [29], Lima & Gerhard [30], Santos & Zanata [31], Birindelli et al. [32], Santos & Caramaschi [33], and Pereira et al. [20].

## Discussion

Twenty two species of *Parotocinclus* are known from the Atlantic coastal basins of northeastern and southeastern Brazil, including the Rio São Francisco basin. The geographically closest species to *P*. *nandae* are *P*. *adamanteus* Pereira, Santos, de Pinna & Reis, 2019, *P*. *arandai*, *P*. *bahiensis*, *P*. *cristatus*, *P*. *doceanus*, *P*. *jimi*, and *P*. *minutus* Garavello, 1977, all from the Northeastern Mata Atlântica freshwater ecoregion, particularly in an area between the Rio Vaza Barris on the north, in Sergipe State, and the Rio Doce on the south, in Espírito Santo State (Fig 7). *Parotocinclus nandae* is morphologically most similar to *P*. *adamanteus*, *P*. *doceanus*, *P*. *jimi*, and *P*. *maculicauda* in a series of features, including the shared standard length similar (max. 60 mm), number of vertebrae (28–30), plates in median lateral series (24–27), and plates between anal and caudal fins (11–12). However, the new species is easily distinguished from these species by a series of features, including its unique color pattern, the lower lip elongated posteriorly reaching or almost reaching to the pectoral girdle, the abdomen with embedded small plates, and by the canal cheek plate with a slightly concave margin, not expanded mesially. Also, *P*. *nandae* differs from all congeners of the Atlantic coastal basins of southeastern and northeastern of Brazil (except *P*. *cesarpintoi* Miranda Ribeiro, 1939 and *P*. *cristatus* Garavello, 1977) by the presence of a tuft of hypertrophied odontodes on the posterior tip of parieto-supraoccipital, by having the branched rays and interradial membranes of the pectoral and pelvic fins unpigmented in ventral view (except *P*. *arandai* Sarmento-Soares, Lehmann A. & Martins-Pinheiro, 2009, *P*. *bahiensis* (Miranda Ribeiro, 1918), *P*. *spilurus* (Fowler, 1941), *P*. *prata* Ribeiro, Melo & Pereira, 2002), by the rostral plate not exposed ventrally and not covering the tip of the snout (except *P*. *adamanteus*, *P*. *arandai*, *P*. *jequi* Lehmann A., Koech Braun, Pereira & Reis, 2013, *P*. *prata*, *P*. *robustus* Lehmann A. & Reis, 2012, *P*. *seridoensis* Ramos, Barros-Neto, Britski & Lima, 2013, and *P*. *spilurus* (Fowler, 1941), with the rostral plate exposed ventrally and visible on snout tip), and by the pectoral girdle covered by thick skin medially and exposed supporting odontodes only laterally, restricted to coracoid (except for *P*. *adamanteus*, *P*. *jequi*, and *P*. *prata*, with the pectoral girdle exposed and supporting odontodes medially and laterally). The new species differs from *P*. *adamanteus*, a recently described and sympatric species in the Rio Capivari, by the absence of a distinct rostral border forming a fleshy intumescence on the lateral portion of the head ornamented with moderately hypertrophied odontodes in adult males; by fewer premaxillary teeth 15–25 (vs. 45–61), by the lower lip longer than wide (vs. lower lip wider than longer), by the incomplete mid-dorsal and mid-ventral series of lateral plates, not surpassing the origin of the adipose fin (vs. surpassing the origin of adipose fin by 3–4 plates), and by fewer mid-dorsal and mid-ventral series of lateral plates (15–16 and 18–20 vs. 20–21 and 21–22, respectively) (Fig 3A and 3B).

*Parotocinclus nandae* is further distinguished from congeners from the Amazon and Orinoco basins and the Guianas coastal drainages (*P*. *amazonensis* Garavello, 1977, *P*. *aripuanensis* Garavello, 1988, *P*. *britskii* Boeseman, 1974, *P*. *collinsae* Schmidt & Ferraris, 1985, *P*. *dani* Roxo, Silva & Oliveira, 2016, *P*. *eppleyi* Schaefer & Provenzano, 1993, *P*. *halbothi* Lehmann A., Lazzarotto & Reis, 2014, *P*. *longirostris* Garavello, 1988, *P*. *polyochrus* Schaefer, 1988, *P*. *variola* Lehmann A., Schvambach & Reis, 2015, and *P*. *yaka* Lehmann A., Lima & Reis, 2018) by having the canal cheek plate rounded not elongated posteriorly and not contacting the pectoral girdle (*vs*. canal plate elongated posteriorly on the ventral surface of head and contacting the cleithrum).

The presence of a thick and rough skin in the interradial membrane of pelvic fin exclusively in the females of *P*. *nandae* (Fig 4) is a feature reported by the first time to occurs in Siluriformes. The cellular composition and function of this feature are unknown, but it may be related to the production of pheromone. Reis (1998, Ch. 59: 132) mentioned the presence of a somewhat similar thick adipose body on the ventral surface of the pectoral fins of mature males of some Callichthyidae genera. According to the author, the thick tissue appears only during the reproductive season and may be related to the production of pheromone. Further studies are needed to better understand the remarkable feature present in the pelvic fin of females of *P*. *nandae* and to check its occurrence within other hypoptopomatins.

### Additional specimens

*Parotocinclus adamanteus*: Brazil, Bahia. MZUSP 120470, 2, 42.0–42.0 mm SL; Lençóis, Ribeirão de Baixo, tributary of Rio São José, tributary of Rio Paraguaçu, 12°39'32"S 41°22'12"W. MZUSP 120499, 2, 32.7–32.9 mm SL; Palmeiras, Rio Conceição, tributary of Rio Preto, locality of Conceição dos Gatos, Chapada Diamantina. Rio Paraguaçu basin. 12°32'33"S 41°31'13"W. MZUSP 120503, 5, 18.7–36.2 mm SL; Palmeira, upper Rio Preto, tributary of Rio Paraguaçu, Vale do Capão, Chapada Diamantina, 12°36'38.0"S 41°30'49.2"W. Paratypes: MCP 54151, 4, 33.0–53.8 mm SL + 3 c&s, 41.4–44.5 mm SL, Lençóis, Ribeirão de Baixo near mouth into Rio São José, Rio Paraguaçu basin (12°35’11”S 41°22’58”W); MCP 54160, 1 c&s, 51.5 mm SL, Andaraí, Rio Garapa, tributary to Rio São José approx. 600 m upstream road from Lençóis to Andaraí, Rio Paraguaçu basin. 12°44’44”S 41°20’44”W.
